# Single-Cell Atlas of the Chinese Tongue Sole *(Cynoglossus semilaevis)* Ovary Reveals Transcriptional Programs of Oogenesis in Fish

**DOI:** 10.3389/fcell.2022.828124

**Published:** 2022-03-01

**Authors:** Xiang Liu, Yingyi Huang, Fujian Tan, Hong-Yan Wang, Jian-Yang Chen, Xianghui Zhang, Xiaona Zhao, Kaiqiang Liu, Qian Wang, Shanshan Liu, Francesc Piferrer, Guangyi Fan, Changwei Shao

**Affiliations:** ^1^ College of Fisheries and Life Science, Shanghai Ocean University, Shanghai, China; ^2^ Key Lab of Sustainable Development of Marine Fisheries, Ministry of Agriculture and Rural Affairs, Yellow Sea Fisheries Research Institute, Chinese Academy of Fishery Sciences, Qingdao, China; ^3^ BGI-Qingdao, BGI-Shenzhen, Qingdao, China; ^4^ BGI-Shenzhen, Shenzhen, China; ^5^ Laboratory for Marine Fisheries Science and Food Production Processes, Qingdao National Laboratory for Marine Science and Technology, Qingdao, China; ^6^ Qingdao-Europe Advanced Institute for Life Sciences, BGI-Shenzhen, Qingdao, China; ^7^ College of Marine Technology and Environment, Dalian Ocean University, Dalian, China; ^8^ School of Marine Sciences, Ningbo University, Ningbo, China; ^9^ Institut de Ciències Del Mar (ICM), Spanish National Research Council (CSIC), Barcelona, Spain

**Keywords:** scRNA-seq, oogenesis, Chinese tongue sole, germ cell, granulosa cell

## Abstract

Oogenesis is a highly orchestrated process that depends on regulation by autocrine/paracrine hormones and growth factors. However, many details of the molecular mechanisms that regulate fish oogenesis remain elusive. Here, we performed a single-cell RNA sequencing (scRNA-seq) analysis of the molecular signatures of distinct ovarian cell categories in adult Chinese tongue sole (*Cynoglossus semilaevis*). We characterized the successive stepwise development of three germ cell subtypes. Notably, we identified the cellular composition of fish follicle walls, including four granulosa cell types and one theca cell type, and we proposed important transcription factors (TFs) showing high activity in the regulation of cell identity. Moreover, we found that the extensive niche–germline bidirectional communications regulate fish oogenesis, whereas ovulation in fish is accompanied by the coordination of simultaneous and tightly sequential processes across different granulosa cells. Additionally, a systems biology analysis of the homologous genes shared by Chinese tongue sole and macaques revealed remarkably conserved biological processes in germ cells and granulosa cells across vertebrates. Our results provide key insights into the cell-type-specific mechanisms underlying fish oogenesis at a single-cell resolution, which offers important clues for exploring fish breeding mechanisms and the evolution of vertebrate reproductive systems.

## Introduction

Oogenesis is a markedly complex, dynamic process, from self-renewal and differentiation of oogonia, development of follicle cells, maturation of oocytes and ovulation (Gershon and Dekel, 2020). Natural selection ensures that the structure and function of each organism provides, under the environment that organism has adapted to, the best conditions for the survival of its germ cells. The transition from oviparity to viviparity is a crucial evolutionary change in the life history of vertebrates. The main reproductive mode of teleosts is oviparity with lecithotrophy, i.e., zebrafish, sea bass and tongue sole, while most mammals show viviparity with matrotrophy, i.e., human, monkey and mouse (Lodé, 2012). However. it is barely known whether vertebrates with different reproductive modes retain the essential transcription characteristics of oogenesis in evolution. At least within mammals, previous research showed that human oogenesis appears to be more complex than oogenesis in other mammals (Sylvestre et al., 2013; Biase, 2017); however, shared gene expression patterns between human and mouse oocytes suggest some evolutionarily conserved mechanisms of mammalian oogenesis (Zhang et al., 2018).

During oogenesis, the development of germ cells is accompanied by the transition from mitosis to meiosis and the expression of stage-specific genes (Zhao et al., 2020b). The dynamic translation of the germ cell state is regulated by stage-specific transcription factors (TFs). In humans, TFs such as *GTF2l*, *CSDE1*, *SOHLH2*, *SMARCE1,* and *TUB* are considered to play a crucial role in the transition from the primordial to the primary stage (Zhang et al., 2018). In addition, several TFs, such as *Msx1*, *Msx2*, *Gata4*, *Cdx2,* and *Sox4*, have strong effects on the transition from mitosis to meiosis in mouse oogenesis (Zhao et al., 2020b). Nevertheless, the TFs that participate in fish oogenesis have rarely been explored or identified, despite the enormous implications of the proper regulation of fish oogenesis for both fisheries and aquaculture production.

Each follicle is composed of a germ cell surrounded by follicle cells (Sánchez and Smitz, 2012). Follicle cells regulate the maturation and ovulation of oocytes through complex interactions and endocrine processes (Li and Albertini, 2013). In female mammals, follicle cells are anatomically divided into mural granulosa cells and cumulus cells (Wigglesworth et al., 2015). Additionally, some investigators have identified the transcription characteristics of mural granulosa cells and cumulus cells as well as associated signals regulating oogenesis at the single-celllevel (Fan et al., 2019; Zhao et al., 2020a). The BMP15/GDF9 and NOTCH signaling pathways are thought to be two key pathways that regulate oogenesis (Eppig et al., 1997; Zhang et al., 2011). In fish, light and electron microscopy analyses have revealed a follicular complex consisting of a mature oocyte, zona radiata, follicle cells, basement membrane and theca (Viana et al., 2018). However, the subtypes of fish follicle cells and the regulation in oogenesis are not systematically understood.

Another key developmental event in oogenesis is ovulation, which is the process whereby mature oocytes break away from the surrounding somatic cells (granulosa cells and theca cells) under a luteinizing hormone (LH) surge (Curry, 2010). Many investigations of human oogenesis have demonstrated that proteinases such as plasminogen activators, matrix metallopeptidase and a disintegrin and metalloproteinase with thrombospondin type 1 motif (ADAMTS) mediate cumulus oocyte complex (COC) expansion and oocyte release by hydrolysing extracellular matrix (ECM) proteins in the follicular layer (Curry, 2010). Notably, prostaglandins (PGs) are an important signaling molecule involved in ovulation in mammals and teleosts (Takahashi et al., 2018). However, the ovulation mechanism of other vertebrates is still lack of in-depth research, especially in fish.

Chinese tongue sole (*Cynoglossus semilaevis*) is a flatfish with asynchronous oocyte development and batch spawning since oocytes of diverse developmental stages are found in the ovary in the same growth phase (Chen et al., 2010; Wen et al., 2015), which lends itself to the investigation of fish oogenesis. The whole-genome sequence of Chinese tongue sole was completed in 2014 (Chen et al., 2014), which provides a foundation for the transcriptomic researches. In the present study, we performed single-cell RNA sequencing (scRNA-seq) to analyse the key events throughout oogenesis in Chinese tongue sole. We described the progression of germ cells and transcriptional characteristics of somatic niche cells. Importantly, we identified diverse granulosa cell subtypes and inferred the key highly active TFs involved in follicle cell fate decisions. Moreover, we found that the niche–germline bidirectional communications regulate fish oogenesis, whereas the interaction between different granulosa cells contributes to the ovulation of fish. Furthermore, by cross-species comparison of ovarian cells from Chinese tongue sole and macaques, we revealed the conserved gene expression features in oogenesis across mammals and fish. Our results provide a solid reference dataset for research on fish propagation-related biology and sheds light on the adaptability of reproductive strategies in lower vertebrates.

## Materials and Methods

### Hematoxylin-Eosin Staining of Chinese Tongue Sole Ovary

To make paraffin sections, the ovary tissue was fixed in the fixing agent for at least 24 h. For dehydration and paraffin embedding, samples were placed in 75% ethanol for 4 h, 85% ethanol for 2 h, 90% alcohol for 2 h, 95% ethanol for 1 h, 100% ethanol I for 30 min, 100% ethanol II for 30 min, ethanol benzene for 5–10 min, xylene II for 5–10 min, 65°C melting paraffin I for 1 h, 65°C melting paraffin II for 1 h, and 65°C melting paraffin III for 1 h. The wax-soaked tissue was embedded in an embedding machine. Samples were cooled at −20°C on a freezing table. After the wax solidified, the wax block was removed from the embedding frame and repaired. The trimmed wax block was sectioned on a microtome paraffin slicer with a thickness of 4 μm. For Hematoxylin-eosin staining, dewaxing was performed by rinsing in xylene I for 20 min, xylene II for 20 min, 100% ethanol I for 5 min, 100% ethanol II for 5 min, and 75% ethanol for 5 min. Sections were then rinsed with tap water, stained with hematoxylin solution for 3–5 min, and rinsed with tap water. Then, the sections were treated with Hematoxylin Differentiation solution and rinsed with tap water. The sections were treated with Hematoxylin Scott Tap Bluing and rinsed with tap water. They were then treated with 85% ethanol for 5 min and 95% ethanol for 5 min. Finally, sections were stained with Eosin dye for 5 min. For dehydration, sections were treated with 100% ethanol I for 5 min, 100% ethanol II for 5 min, 100% ethanol III for 5 min, xylene I for 5 min, and xylene II for 5 min, followed by sealing with neutral gum. Sections were observed by microscopy and images were obtained for analysis.

### Dissociation of Ovarian Cells in Female Chinese Tongue Sole

To obtain a high-quality cell suspension, we strictly controlled the preparation time. First, six adult female Chinese tongue sole were dissected and their ovaries were removed. The ovaries of Chinese tongue sole were dissected on ice and washed twice with DMEM-NaCl solution, and other tissues that adhered to the ovaries were removed with tweezers. Then, the combined enzyme digestion solution was prepared. Add 1 ml of trypsin with a concentration of 0.25% and 200 μL of collagenase with a concentration of 1 mg/ml to 3.8 ml of DMEM-NaCl solution. The ovarian tissue was cut with a blade to produce a tissue homogenate, which was then transferred to a combined enzyme digestion solution. After heating in a 30°C water bath for 7 min, digestion was rapidly terminated with 10 ml DMEM-NaCl solution. The digested cell suspension was filtered with a 40 μm cell strainer to collect approximately 7 ml of the filtered cell suspension in a 15 ml tube. Centrifugation was performed with a horizontal rotor in a cryogenic centrifuge at 300 × *g* for 3 min. After removing the supernatant, 5 ml of PBS-NaCl solution was added, centrifugation was conducted at 300 × *g* for 3 min, and washing was performed twice. Finally, 0.05% BSA in PBS was added to resuspend the cells. Finally, 10 μL single-cell suspensions was mixed with 1 μL 0.4% dye trypan blue solution to measure the total cell concentration (1,000–2000 cells per 1 μL) as well as the ratio of live cells (greater than 80 percent) using a hemocytometer.

### scRNA-Seq Library Preparation and Sequencing

We used the DNBelab C Series Single Cell RNA Library Preparation Kit (MGI Tech Co., Ltd.) based on droplet microfluidics technology for library construction. In detail, we pooled 110,000 cells per library with an average concentration of 1,000 cells/μL and activity greater than 80%. Cells were prepared as droplets, in which cell lysis and mRNA capture were performed using the DNBelab C4 portable single-cell system. Single-cell microdroplets were recovered by using the emulsion breaking recovery system, after which magnetic bead-captured mRNA was transcribed into cDNA and subjected to reverse transcription (42°C for 90 min; 14 cycles of 50°C for 2 min and 42°C for 2 min; holding at 4°C) for cDNA enrichment. Finally, the cDNA products were used to prepare single-stranded DNA libraries via steps including shearing, end repair, ligation, PCR (95°C for 3 min; 11 cycles of 98°C for 20 s, 58°C for 30 s and 72°C for 30 s; 72°C for 10 min; holding at 4°C), denaturation, circularization, and digestion. Then, 10 ng of the digested products was collected for sequencing on the MGISEQ 2000 platform.

### scRNA-Seq Data Processing

The raw data obtained by scRNA-seq on the MGISEQ2000 platform were filtered and demultiplexed using PISA (version 1.10.2) (https://github.com/shiquan/PISA). Reads were aligned to the reference genome from NCBI using STAR (version 2.7.9a) (https://github.com/alexdobin/STAR) and were sorted using Sambamba (version 0.7.0) (https://github.com/biod/sambamba/releases/tag/v0.7.0). The cell versus gene UMI count matrix was generated using PISA.

### Cell Clustering and Identification of Cell Types

For each library, we used the count matrix and followed the Seurat vignette (https://satijalab.org/seurat/pbmc3k_tutorial.html) to create the Seurat object. In brief, cells were first prefiltered according to the expression of at least 200 genes in each cell and the expression of each gene expressed in at least 3 cells. Thereafter, cells with less than 5% mitochondrial genes and UniQ gene counts between 200 and 3500 were retained. For the remaining cells, the gene expression count data for each sample were normalized, 3,000 highly variable genes were selected, and scaling was conducted with the “SCTransform” function. After normalization, principal component analysis (PCA) was used to reduce the number of dimensions of the gene expression matrix. The number of components used was determined with the “ElbowPlot” function. The first 30 PCs were selected as the input for the “FindNeighbors” and “RunUMAP” functions (default parameters), and a parameter of resolution of 0.5 was chosen for the “FindClusters” function. Subsequently, “DoubletFinder” was employed to identify doublets using the same PCs of the above PCA, assuming a 4% or 10% doublet formation rate for the loaded cells of each sample in a droplet channel. The optimal pK values were determined for each organ based on the mean-variance normalized bimodality coefficient. After doublet removal of all library, we merge the six matrices and use the “selectintegrationfeatures” function to select the top 3,000 genes with the greatest variation for integration, after which we used the “prepsctintegration” function to aggregate SCT objects, and then the “findintegrationanchors” function with the normalization. method parameter as “SCT”, the top 3,000 genes as the anchor. features and k. filter parameter as 200 to identify the sources of common variation between conditions via canonical correlation analysis (CCA). This procedure was conducted find the anchor or nearest neighbour (MNN) for cross-dataset identification, after which the anchor points were filtered, incorrect anchor points were removed, and the data were finally integrated by using the “integration data” function with the normalization. method parameter as “SCT”. After integrating and removing batch effects, we ran the PCA (30PCs) and UMAP (default parameters) analyses again, and we finally used the “findallmarker” function (with only. pos equal to TRUE, min. pct and logfc. threshold equal to 0.25 and test. use equal to “wilcox”)to calculate the DEGs between different clusters.

### Gene Function Enrichment Analysis

Gene function enrichment analysis was performed using Metascape (https://metascape.org/gp/index.html). The Chinese tongue sole genes used for the enrichment analysis were homologous to zebrafish genes.

### Cell Trajectory Analysis

Single-cell pseudotime trajectories were constructed with the Monocle 2 package (version 2.14.0) (http://cole-trapnell-lab.github.io/monocle-release/docs/). In describing the process of oogenesis, we transformed the Seurat dataset into the cell_data_set of Monocle2. Then, we chose the ordered genes to define cell progress, we used DDRTree to reduce the space to two dimensions, and all cells were ordered with the orderCells function with the default parameters.

### Identification of DEGs Between Two Different Clusters

The DESeq2 (v1.30.1) R package was used to find additional DEGs. The package uses non-normalized counts as an input. The data from each sample were aggregated, and the *DESeqDataSetFromMatrix* function was used to construct the count matrix. *DESeq* and the *results* function were used for the differential expression analysis and to generate results tables.

### Transcriptional Regulation Analysis of Follicle Cell Subtypes

The SCENIC analysis was run as described on the follicle cell subclusters, using the 10-thousand motifs database for RcisTarget and GENIE3. The input matrix was the normalized expressional matrix, oriented from the one-to-one homologous genes of human and Chinese tongue sole.

### Cytokine/Receptor Interaction Analysis

ITALK was used to analyse the cell-cell communication between three germ cell subtypes and five granulosa cells subtypes. Firstly, we transformed the matrix of tongue sole into the matrix of human. The one-to-one homologous genes of human and Chinese tongue sole were obtained by blast. Then we set the matrix and cell types as the input data, and the ligand-receptor pairs were detected from top 50% highly expressed genes. We set growth factors, cytokines, checkpoint and other as the communication types. Finally, we using LRPlot function to visualize the network plot.

### Conservation of Marker Gene Expression

Macaque UMI matrices were obtained from the NCBI Gene Expression Omnibus (GEO) database (GSE149629). Germ and somatic cells were extracted from the matrix according to the barcode of the cell type reported previously (Zhao et al., 2020a).

## Results

### The Global Transcriptome Profile Identifies Eight Cell Types in Chinese Tongue Sole Ovaries

**FIGURE 1:**
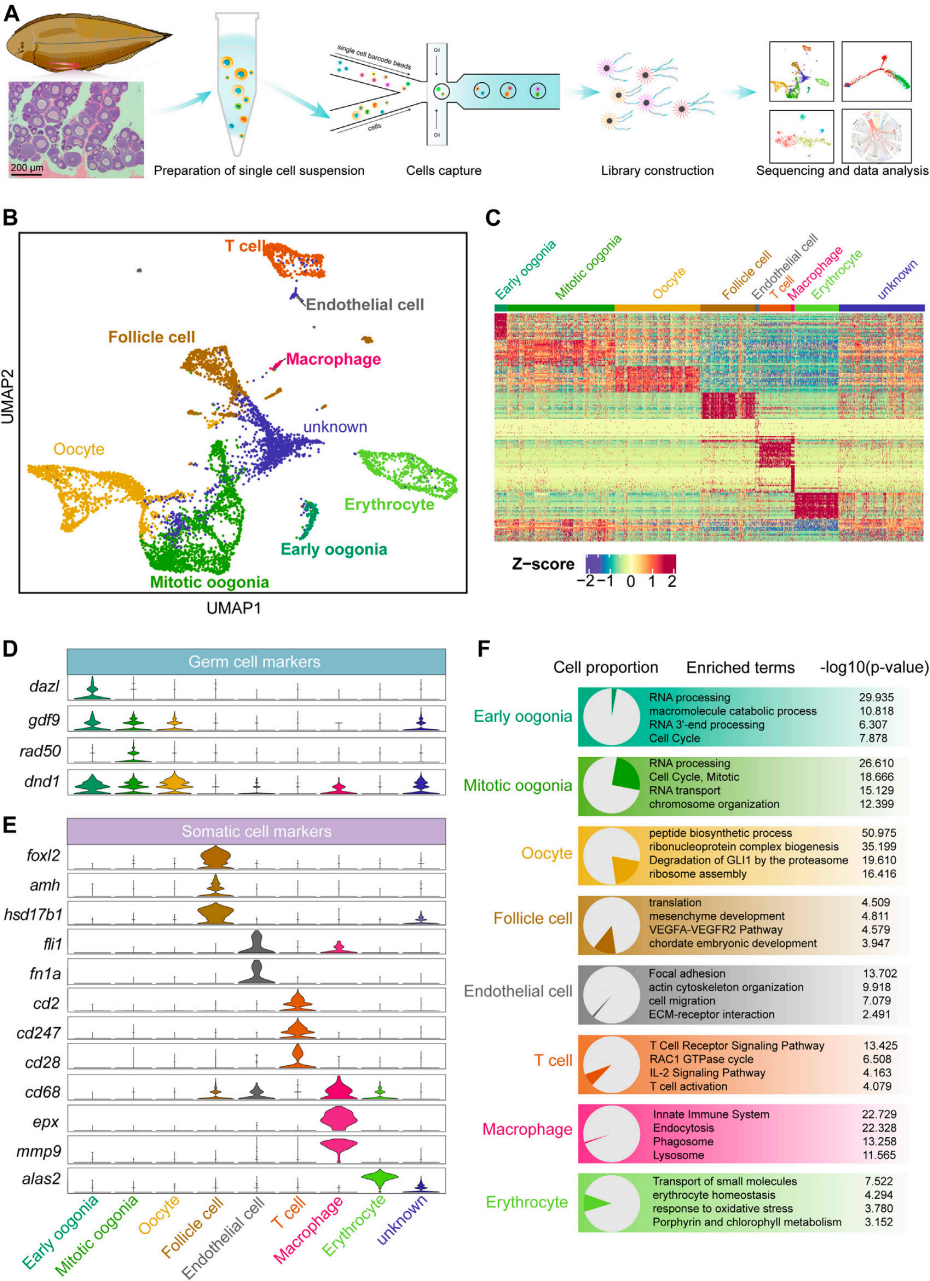
Overview of germ and somatic ovarian cell types determined by scRNA sequencing in Chinese tongue sole. **(A)** Schematic representation of the scRNA-seq analysis of Chinese tongue sole ovarian cells. **(B)** Visualization of major ovarian cell types among 7,185 cells in UMAP (unknown is undefined). Different cell types are shown in distinct colours. **(C)** Heatmap showing the expression of the top 20 DEGs of the main cell types. The colours from blue to red indicate low to high gene expression levels, respectively. Z-scores were calculated by subtracting the average value for the set of data from the value for each cell and dividing by the standard deviation. **(D)** Violin plots of the normalized expression of marker genes in the three germ cell types. **(E)** Violin plots of the normalized expression of marker genes in the five somatic cell types. **(F)** Enriched terms of DEGs are shown for ovarian cell types (*p*-values are shown). The proportion of each cell type is shown on the left.

To capture all the cellular information in the ovary of Chinese tongue sole, we collected the ovaries of six sexually mature females in October. At this time, the ovary was in stage IV and contained oocytes in all stages of development, and the follicles exhibited a double-layer membrane structure. Using the iDrop system, six libraries were constructed and sequenced (Figure 1A). After quality control, we obtained the transcriptional spectrum of 7,185 cells. Using mutual nearest neighbour methodology, we identified 15 clusters, which were visualized via Uniform Manifold Approximation and Projection (UMAP) (Supplementary Figure S1A). Our data showed no apparent batch effect, and each sample contained all clusters (Supplementary Figures S1B, S1C).

Three germ cell populations and five somatic cell populations were identified by gene expression pattern analysis (Figures 1B,C). Typical marker genes of oogenesis, such as *gdf9* and *dnd1* (Liu and Ge, 2007; Yang et al., 2012), were widely expressed in the three identified germ cell types. *Dazl* (Bachvarova et al., 2004; Li et al., 2016; Bertho et al., 2021) was highly expressed in early oogonia, while the mitotic marker *rad50* (Kostyrko et al., 2015; Völkening et al., 2020; Liu et al., 2021) was mainly expressed in mitotic oogonia (Figure 1D). In addition, we identified somatic cells based on the following markers: follicle cells (*foxl2*, *amh* and *hsd17b1*) (Pisarska et al., 2011; Luo et al., 2020; Moolhuijsen and Visser, 2020; Li et al., 2021), endothelial cells (*fli1* and *fn1a*) (Nagai et al., 2018; Wu et al., 2021), T cells (*cd2*, *cd247* and *cd28*) (Hurley et al., 1994; Lundholm et al., 2010), macrophages (*cd68*, *epx* and *mmp9*) (Hunyady et al., 1996; Sendler et al., 2018) and erythrocytes (*alas2*) (Munday et al., 2000; Tsang et al., 2017) (Figure 1E). The top 20 differentially expressed genes (DEGs) in each cell were shown in Supplementary Figure S1D. Furthermore, an enrichment analysis of the DEGs of each cell type was performed based on the Metascape resource. The results showed that “RNA processing” and “cell cycle, mitotic” were enriched in early oogonia and mitotic oogonia, respectively, while “peptide biosynthetic process” was enriched in oocytes. In contrast, follicle cells were enriched in the terms “mesenchyme development”, and endothelial cells were enriched in “focal adhesion” and “ECM-receptor interaction”. In addition, “T cell receptor signaling pathway”, “innate immune system” and “erythrocyte homeostasis” were enriched terms in T cells, macrophages and erythrocytes, respectively (Figure 1F).

### Dynamics of Gene Expression Across Germ Cells Revealed by a Pseudotime Trajectory

**FIGURE 2:**
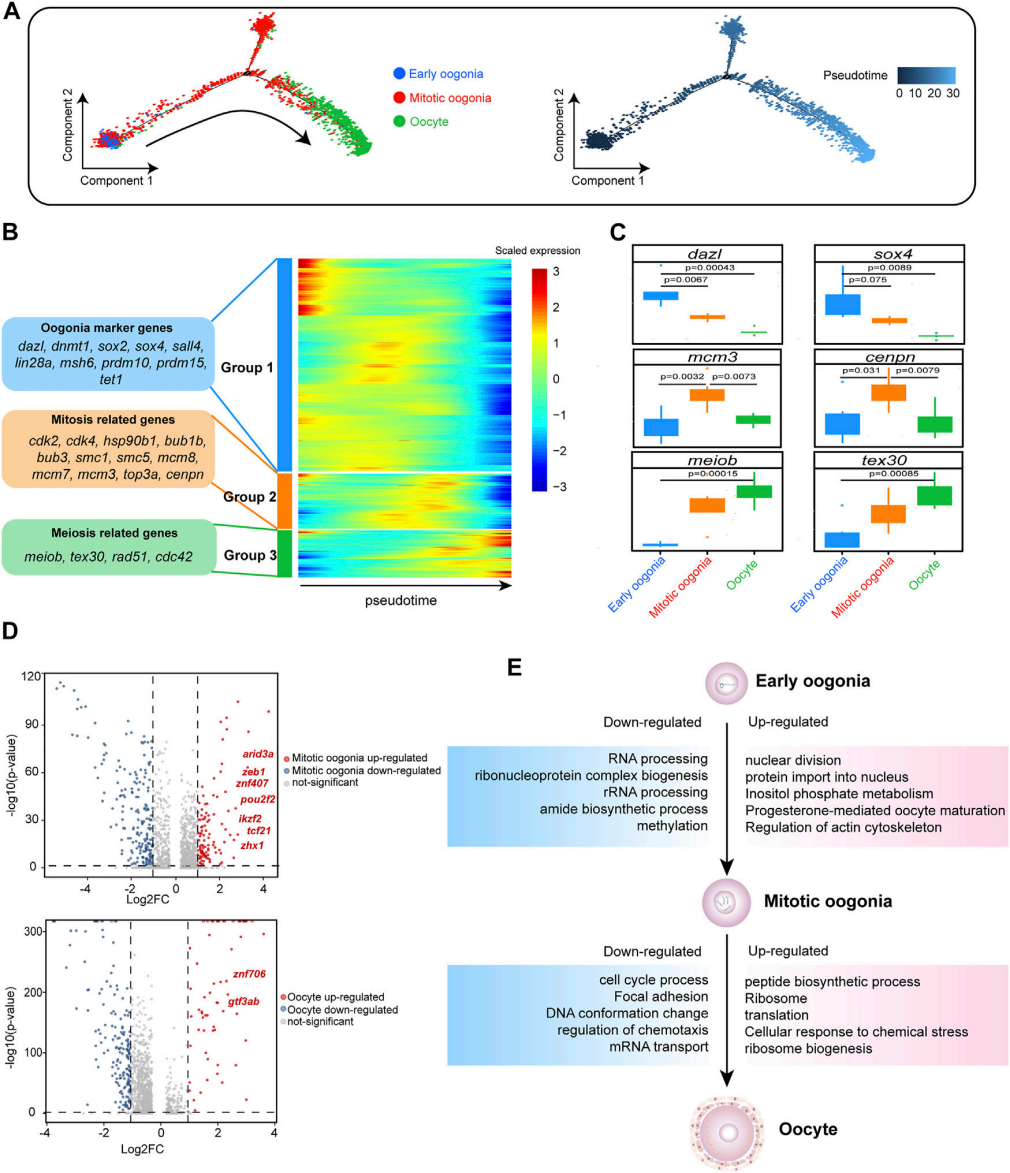
Gene expression dynamics and transcriptional characteristics of ovarian germ cells. **(A)** Developmental pseudotime trajectory of germ cells. Different colours in the figure on the left side represent different cell types. The figure on the right represents the degree of differentiation of the cell types in the pseudotime trajectory (where darker colours indicate a lower degree of differentiation). **(B)** Gene expression heatmap over pseudotime. After clustering analysis, genes were divided into three groups (Groups 1–3) (representative genes are shown on the left). Colours from blue to red indicate low to high gene expression levels, respectively. **(C)** Boxplot showing the mean expression levels of oogenesis-associated genes in each germ cell subtype. Two-tailed *t* test *p*-values are indicated. **(D)** Volcano plot showing the DEGs of germ cells, setting a fold change >2 as the threshold (dotted line). In the upper figure, upregulated genes in mitotic oogonia relative to early oogonia are indicated in red, and downregulated genes are indicated in blue. In the lower figure, upregulated genes in oocytes relative to mitotic oogonia are indicated in red, and downregulated genes are indicated in blue. Upregulated transcription factors (TFs) are labelled. **(E)** DEGs and associated enriched terms (identified using Metascape) characteristic of germ cell developmental transitions, based on Figure 2D. Upregulated terms are annotated in pink boxes, and downregulated terms are annotated in blue boxes.

To explore the development of female germ cells in fish, Monocle2 was employed for cell trajectory analysis of the three identified germ cell types to recapitulate the temporal progression of oogenesis. Notably, in the unsupervised pseudotime analysis, early oogonia, mitotic oogonia and oocytes followed a continuous trajectory. Early oogonia were observed at the beginning of the pseudotime, followed by mitotic oogonia and, finally, oocytes at the end point (Figure 2A). Furthermore, hierarchical clustering analysis of the ordered genes showed three groups with different expression patterns. Many markers of oogonia, such as *dazl*, *dnmt1*, *sox2*, *sox4*, *sall4* and *lin28a*, were enriched in the first group. Moreover, the second group contained many mitosis-related genes, such as *cdk2*, *cdk4*, *hsp90b1*, *bub1b*, *bub3*, *smc1* and *smc5*. The third group showed striking meiotic features, such as *meiob*, *tex30* and *rad51* expression (Figure 2B). This suggests that the cells arranged in pseudotime order undergo a transition from non-division to mitosis and then meiosis. Additionally, we analysed the differential expression of these genes in three germ cell types. As expected, two markers of germ stem cells, *dazl* and *sox4*, were significantly overexpressed in early oogonia. Subsequently, the expression levels of the mitotic markers *mcm3* and *cenpn* significantly decreased during the transition from mitotic oogonia to oocytes, while the expression of meiotic markers, such as *meiob* and *tex30*, increased strikingly (Figure 2C). This was consistent with observations in mice (Zhao et al., 2020b). Taken together, these findings suggest that fish oogenesis is a biological process similar to mammalian oogenesis, in which proliferation via mitosis is followed by meiosis.

The molecular mechanism promoting the state transition of fish germ cells is unclear. Therefore, we conducted a differential analysis of the transcripts of different germ cells. Compared with early oogonia, there were 135 significantly upregulated genes in mitotic oogonia (| log2FC | > 1), including seven TFs (*arid3a*, *znf407*, *zeb1*, *pou2f2*, *ikzf2*, *tcf21* and *zhx1*). In addition, there were 62 significantly upregulated genes in oocytes (| log2FC | > 1) compared with mitotic oogonia, including two TFs (*gtf3ab* and *znf706*) (Figure 2D). We hypothesized that these TFs might play a critical role in the transition of germ cells. Simultaneously, we found that “RNA processing” in early oogonia was replaced by “nuclear division” in mitotic oogonia, and “cell cycle process” in mitotic oogonia was replaced by “peptide biological process” in oocytes according to the enrichment analysis of DEGs (Figure 2E). These results indicated that the dynamic transcriptome regulates fish oogenesis in a stage-specific manner.

### Identification of Follicle Cell Subtypes in Fish Ovaries

**FIGURE 3:**
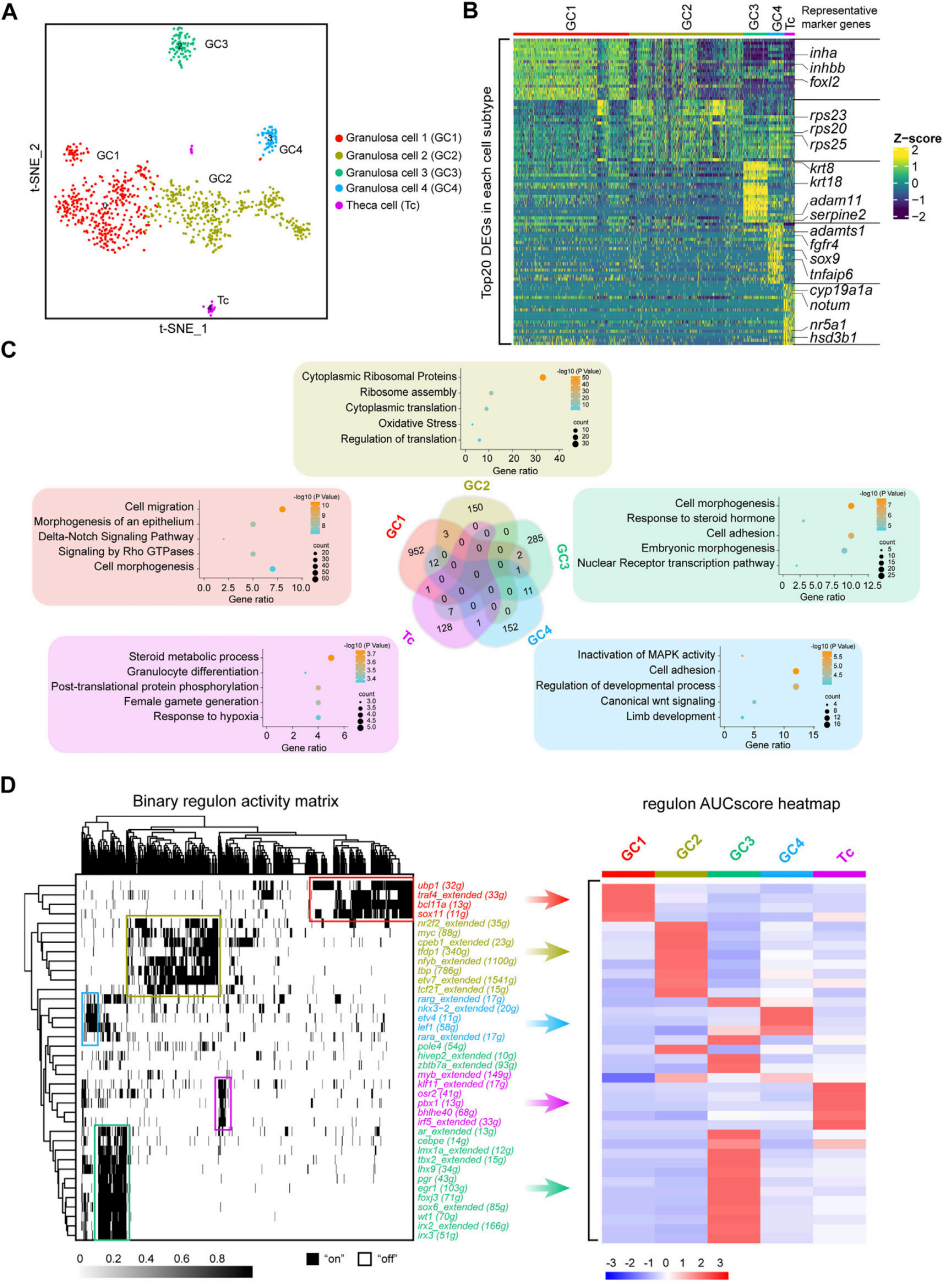
Identification of five discrete transcriptional states of follicle cells. **(A)** UMAP of follicle cell re-clustering. **(B)** Heatmap of the top 20 DEGs in different subclusters of follicle cells. The colours from dark blue to yellow indicate low to high gene expression levels, respectively. Representative marker genes are shown in right. **(C)** Venn diagram showing the intersection of DEGs of the five subclusters. Representative enriched terms for DEGs are shown. **(D)** Left: SCENIC binary regulon activity heatmap depicting follicle cell-enriched regulons. “On” indicates active, while “Off” indicates inactive; Right: Heatmap of the AUC scores of follicle cell subtype-specific regulons. The regulons correspond to the graph on the left.

Ovarian follicle cells play an important role in oocyte maturation and ovulation. Mature fish oocytes are surrounded by two major cell layers, an outer thecal cell layer and an inner granulosa cell layer (Nagahama and Yamashita, 2008; Nagahama et al., 1995; Viana et al., 2018). To explore whether fish follicle cells have different subtypes, similar to those of mammals, we performed a re-clustering analysis of the follicle cells and revealed the existence of four distinct granulosa cell subclusters (GC1, GC2, GC3 and GC4) and one theca cell type (Tc) (Figure 3A). To systematically investigate the similarities and differences between subclusters, we performed a correlation analysis together with the hierarchical clustering of subclusters. This approach highlighted that GC1, GC2, GC3, and GC4 were more closely grouped together, while Tc was different from the other four clusters (Supplementary Figure S2A). To examine the features of the subclusters, we detected the DEGs in each cluster, and the genes with highly variable expression were identified. The results showed that there were significant differences in gene expression among different subclusters (Figure 3B). We found that marker genes of mammalian cumulus cells, such as *foxl2*, *inha* and *inhbb,* were highly expressed in GC1, while marker genes of mammalian mural granulosa cells, such as *krt8* and *krt18,* exhibited peak expression specifically in GC3. Other granulosa cell marker genes such as *adamts1*, *fgfr4*, *sox9*, and *tnfaip6* were specific expressed in GC4. Additionally, we observed that ribosomal protein coding genes were highly expressed in GC2 (Figure 3B). Notably, cell-cycle phase prediction showed that GC2 were more proliferative than other clusters, suggesting that GC2 was a proliferating granulosa cell type (Supplementary Figure S2B). Androgen synthesis related genes such as *nr5a1* and *hsd3b1* were highly expressed in Tc (Figure 3B). It is well established that the theca cells of fish are the source of androgens, which are transferred to granulosa cells where they are converted to oestrogens by the activity of aromatase (Nagahama and Yamashita, 2008).

Subsequently, we analysed the overlap of subclusters of DEGs. Venn diagrams showed that only a small number of DEGs overlapped between each subcluster. To further ascertain the features of the subclusters, we performed gene enrichment analysis of each cluster of specific DEGs. According to the gene enrichment scoring results, the genes expressed in GC1 were enriched in “cell migration” and the “Delta-Notch signaling pathway,” and GC2 was enriched in “cytoplasmic ribosomal proteins”. In addition, “response to steroid hormone” and “cell adhesion” were observed in GC3, while “inactivation of MAPK activity” and “cell adhesion” were enriched in GC4 (Figure 3C). Notably, theca cells were enriched in the terms “steroid metabolic process” and “female gamete generation” (Figure 3C). Collectively, these results indicated that subpopulations of follicle cells play distinct roles in fish oogenesis.

After the recapitulation of the follicle cell subtype transcriptome landscape, we used single-cell regulatory network inference and clustering (SCENIC) to deduce the key highly active TFs involved in follicle cell fate decisions. Distinct follicle cell subtypes were enriched in diverse regulons. For example, GC1 was enriched in regulons such as *ubp1*, *traf4*, *bcl11a,* and *sox11*; GC2 was enriched in regulons such as *nr2f2*, *myc*, *cpeb1,* and *tfdp1*; GC3 was enriched in regulons such as *ar*, *lhx9*, *wt1*, and *irx3*; GC4 was enriched in regulons such as *nkx3-2*, *etv4* and *lef1*; and theca cells were enriched in regulons such as *klf11*, *osr2* and *pbx1* (Figure 3D). These TFs may be the driving genes for the differentiation of different follicle cell subtypes.

### The Multilineage Interactome Network in Fish Oogenesis

**FIGURE 4:**
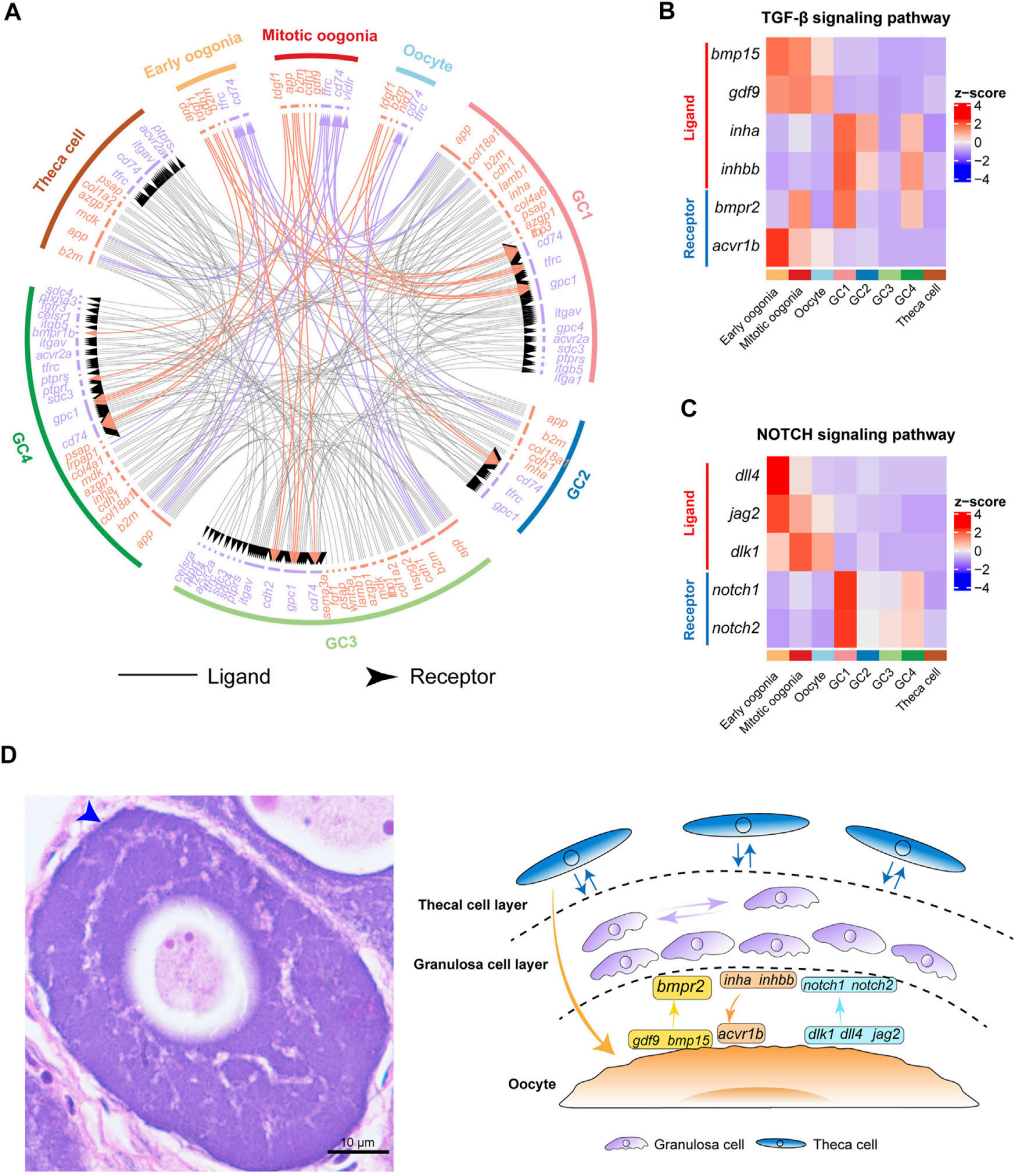
Cell–cell communication networks and signaling pathways involved in fish oogenesis. **(A)** The multilineage interactome network among different cell clusters. Associated interaction pairs are shown. **(B)** Heatmap showing the expression pattern of ligands and receptors of the TGF-β signaling pathway. **(C)** Heatmap shows the expression pattern of ligands and receptors of the NOTCH signaling pathway. **(D)** Left: H&E staining of ovary sections from female Chinese tongue sole. Scale bars represent 10 μm. The blue arrow shows double cell layers of follicle. Right: Schematic summary of interactions involved in fish oogenesis.

To investigate the complex interactions involved in fish oogenesis, we performed an unbiased ligand–receptor interaction analysis between the germ and somatic cell subclusters by using iTALK (Wang et al., 2019). We found that bidirectional communication occurred between germ cells and granulosa cells, while germ cells and theca cells only showed unidirectional regulation from theca cells to germ cells. Additionally, we noted that extensive interactions occurred in distinct somatic cells but no interactions were found between germ cells (Figure 4A). These findings indicate the niche–germline bidirectional communication is mandatory for the development of germ cells and granulosa cells in fish ovaries. The development of theca cells is more dependent on granulosa cells than on germ cells.

Furthermore, we analysed the expression of ligands and receptors of key cell signaling pathways, including the TGF-β and NOTCH signaling pathways. Interestingly, the results showed that the expression of *gdf9* and *bmp15* exhibited peak expression in germ cells (Figure 4B), whereas their receptor *bmpr2* was expressed in both germ cells and granulosa cells. This is consistent with the results observed in human ovaries (Zhang et al., 2018). In contrast, the *inha* and *inhbb* ligands were highly expressed in GC1, whereas their receptor, *acvr1b*, exhibited peak expression specifically in oogonia, highlighting the role of TGF-β signaling in the control of granulosa cell-mediated oogonial proliferation (Figure 4B). These findings suggest that fish oogenesis is regulated by TGF-β signaling, which could be initiated by either germ cells or somatic cells.

Subsequently, we analysed the expression of key components of the NOTCH signaling pathway in germ cells and somatic cells. We found that the *dlk1*, *dll4,* and *jag2* ligands of the NOTCH signaling pathway were predominantly expressed in germ cells. Their receptors, *notch1* and *notch2*, were highly expressed in GC1 (Figure 4C). These results indicate that NOTCH signaling is an important signal that mediates the regulation of granulosa cell proliferation and differentiation by germ cells during fish oogenesis (Figure 4D).

### Ovulation in Fish Is Accompanied by the Synergistic Actions of Different Follicle Cells

**FIGURE 5:**
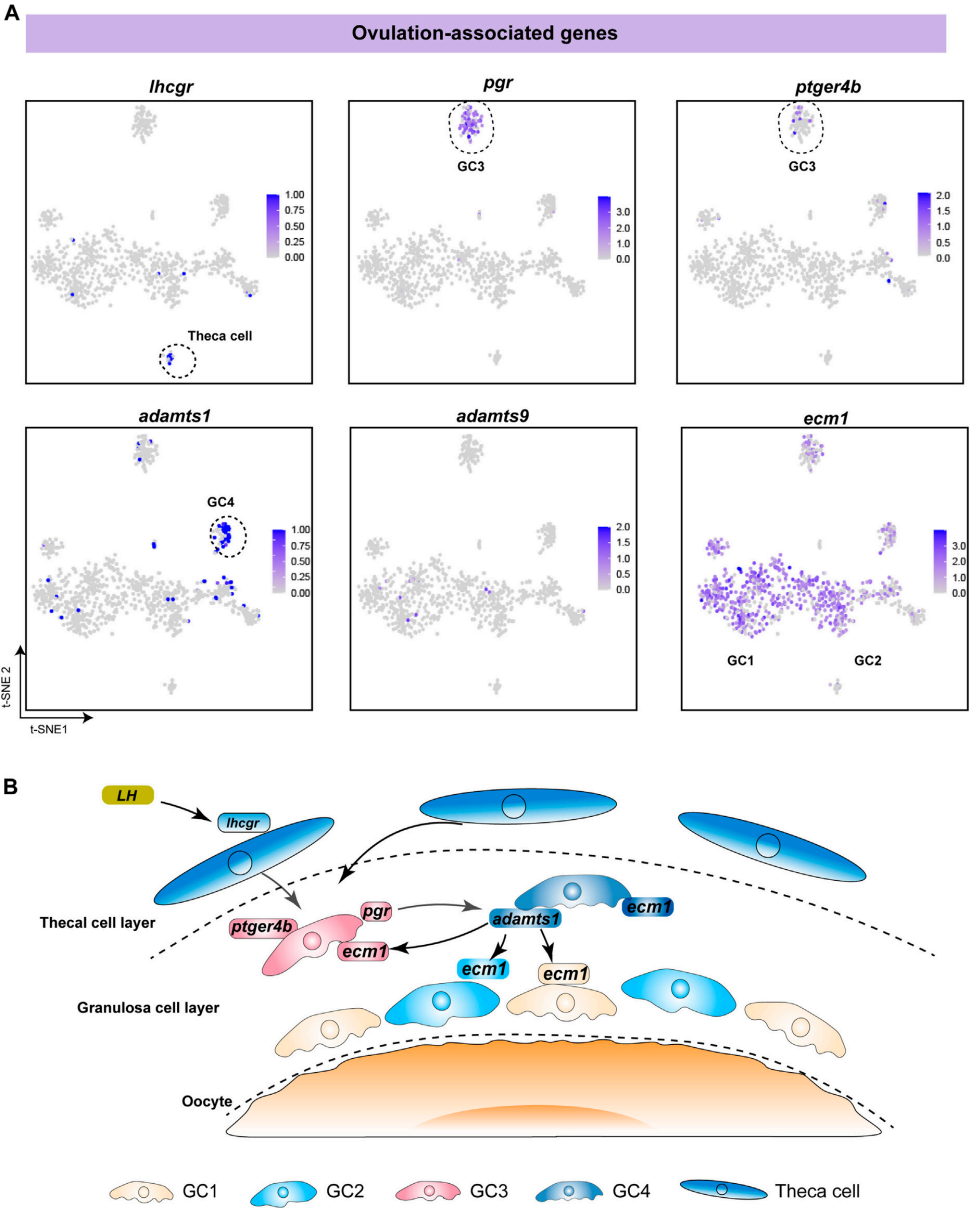
Analysis of specific expression patterns of ovulation-associated genes. **(A)** Feature plot of ovulation-associated genes in different follicle cells. The darker the colour, the higher the expression. Black dashed lines indicate the boundaries of the main clusters of interest. **(B)** Schematic diagram showing the regulatory mechanism of ovulation, involving the synergistic action of different follicle cells in fish.

To investigate the functions of different follicle cell subtypes in fish ovulation, we analysed the expression of pivotal genes in ovulation. Our data showed that *lhcgr*, the LH receptor, was specifically expressed in theca cells, indicating that theca cells receive and transmit LH stimulation during ovulation (Figure 5A). This is consistent with previous studies in mammals and fish (Curry, 2010; Takahashi et al., 2018). Additionally, *pgr* and *ptger4b*, the receptors of progesterone and prostaglandin E2, were highly expressed in GC3, suggesting that progesterone and prostaglandin mainly affect fish ovulation through GC3 (Figure 5A). *Adamts1* and *adamts9* have been reported to be associated with follicle rupture in mammals, especially *adamts1*, which is a protein lyase proven to play an important role in ovulation in a gene knockout experiment (Shozu et al., 2005; Brown et al., 2006). We found that *adamts1* was predominantly expressed in GC4, whereas *adamts9* was expressed in only a few cells (Figure 5A). This suggests that *adamts1* secreted by GC4 might promote follicular rupture during fish ovulation. Notably, *ecm1* was expressed in all subtypes, indicating that *adamts1* acts on the extracellular matrix proteins of distinct granulosa cells to induce different biological changes in the follicle wall (Figure 5A). Taken together, our results suggest that fish ovulation is based on the synergistic action of diverse granulosa cells and theca cells (Figure 5B).

### Comparison of Germ Cell and Granulosa Cell Transcriptomic Profiles Across Species

**FIGURE 6:**
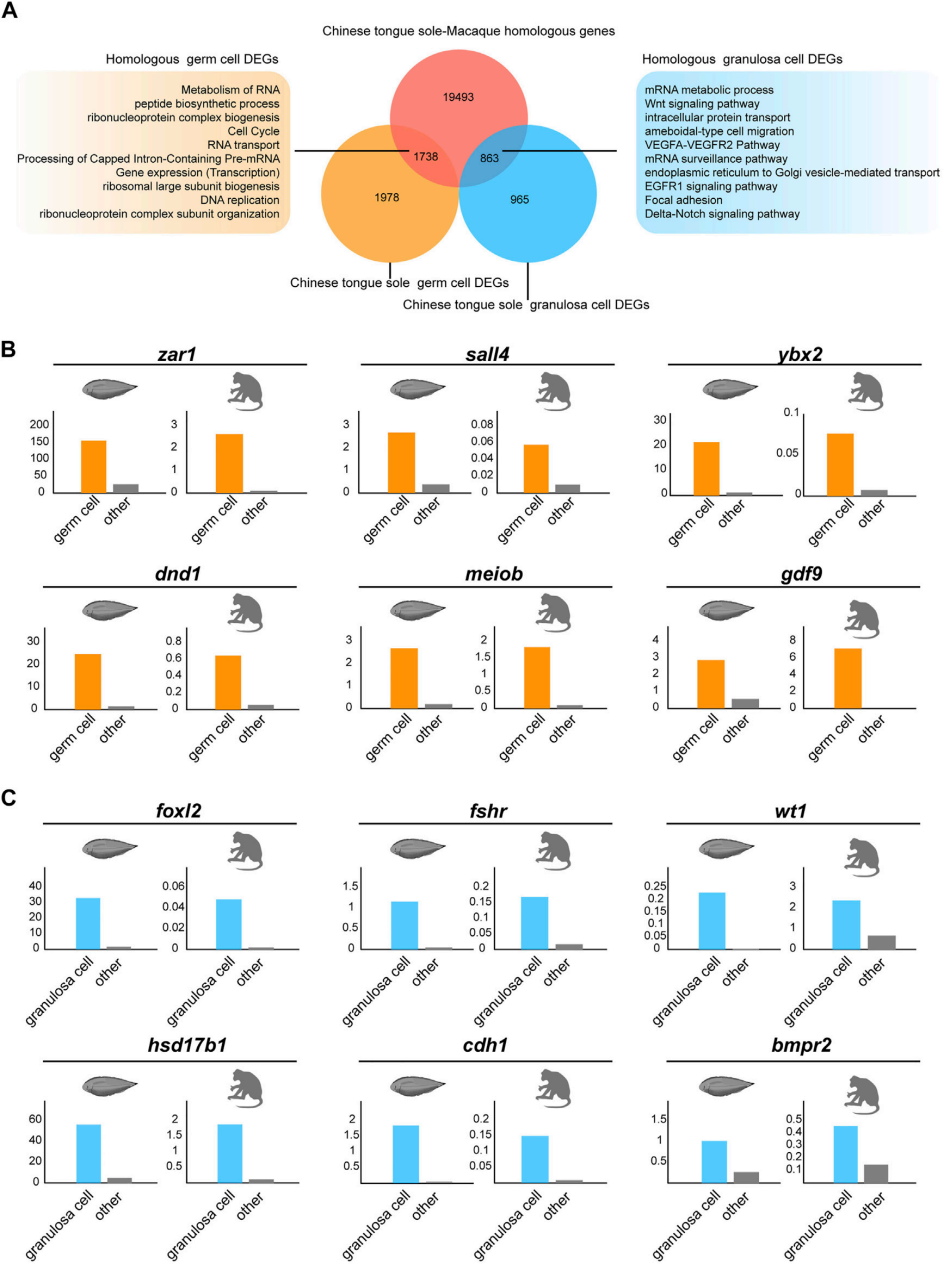
Comparison of germ cells and granulosa cells based on Chinese tongue sole and macaque transcriptomic profiles. **(A)** Venn diagram of DEGs in the germ cells and granulosa cells of Chinese tongue sole showing overlap with the Chinese tongue sole-macaque homologous genes (light red). The enriched terms of the homologous DEGs in germ cells and granulosa cells are shown. **(B)** Histogram of the normalized expression of the shared homologous genes between Chinese tongue sole and macaques related to oogenesis in germ cells relative to the average expression of the same genes in all other cell types. **(C)** Histogram of the normalized expression of the shared homologous genes between Chinese tongue sole and macaques related to oogenesis in granulosa cells relative to the average expression of the same genes in all other cell types.

To better understand the interspecies complexity of germ cell and granulosa cell transcriptomes in fish and mammals, we compared our data with the reported scRNA-seq datasets in macaques (Zhao et al., 2020a). To exclude the effects of abiotic factors, the raw data of the macaques and Chinese tongue sole were analysed via the same procedure. First, utilizing BLASTP, we identified 19,493 homologous genes shared by Chinese tongue sole and macaques. Focusing on germ cells, we identified 1,987 DEGs in Chinese tongue sole. Our results showed that there were 1738 genes with homologs in Chinese tongue sole and macaques (Figure 6A). Subsequently, 965 DEGs of Chinese tongue sole granulosa cells were analysed, 836 of which were homologous. Moreover, the homologous DEGs of the germ cells were enriched in the “metabolism of RNA”, “peptide biosynthetic process” and “cell cycle” terms, while the homologous DEGs of the granulosa cells were enriched in the “Wnt signaling pathway,” “VEGFA-VEGFR2 pathway,” “EGFR1 signaling pathway” and “focal adhesion” and “Delta-Notch signaling pathway” terms (Figure 6A). Notably, these terms are intimately associated with oogenesis. Additionally, several homologous DEGs that have been implicated in oogenesis, such as *zar1*, *sall4*, *ybx2*, *dnd1*, *meiob*, *gdf9*, *foxl2*, *fshr*, *wt1*, *hsd17b1*, *cdh1* and *bmpr2*, showed similar expression patterns in Chinese tongue sole and macaque ovarian cells, suggesting the possibility that they have homologous functions in oogenesis (Figures 6B,C). Thus, these results demonstrate the evolutionarily conserved mechanisms involved in oogenesis across fish and mammals.

## Discussion

Studies on fish reproduction assist the conservation of germplasm resources and the investigation of animal reproductive regulation mechanism in fish model. Most studies on fish oogenesis focus on the structural changes during oocyte maturation observed by light and electron micrograph sections and the regulation of endocrine factors (Selman et al., 1993; Tyler and Sumpter, 1996; Patiño et al., 2005; Kaviani et al., 2013). Conventional genome analysis approaches have been used to capture global gene expression patterns and chromatin conformation of fish ovaries or oocytes (Shao et al., 2014; Can et al., 2020; Dong et al., 2021). However, the bulk sequencing method does not account for cell-to-cell heterogeneity. The development of scRNA-seq technology in recent years can separate cells with different gene expression characteristics through principal component analysis rather than artificial definition, which can overcome these limitations (Tang et al., 2009; Kobak and Berens, 2019). Several studies have revealed unique transcriptional features and gene signatures of oogenesis across vertebrate and invertebrates with single-cell technology (Fan et al., 2019; Rust et al., 2020; Wang et al., 2020; Zhao et al., 2020b). The single-cell transcriptomic atlas of Asian sea bass has identified the specific gene signatures of granulosa cells and oocytes in the fish ovary, that is the first comprehensive analysis of fish ovary at single-cell resolution (Liu et al., 2021). Still, we are far from understanding the dynamic molecular regulation of germ cell development in fish oogenesis, the complex interactions between germ cells and somatic cells, and the transcriptional characteristics of specific cell populations. Thus, herein, we performed a single-cell survey of oogenesis in Chinese tongue sole. We identified eight ovarian cell types by using the known landmark genes and uncovered that pluripotency-related genes, mitosis-related genes and meiosis-related genes were dynamically expressed from oogonia to oocytes. When evaluating the different populations of somatic cells, we defined four granulosa cell subtypes and one theca cell type with diverse signature genes and TFs, which broke through the previous observations of the bilayer follicle cells of fish follicles. Moreover, we explored the bidirectional communication between germ cells and granulosa cells during fish oogenesis, and revealed the interaction of distinct granulosa cell subtypes in fish ovulation through the expression pattern of ovulation-associated genes. Besides, elucidating the conservation and divergence of oogenesis across vertebrates will help us to understand the advantages and disadvantages of various reproductive strategies from different angles and conduct the choice of infertility therapeutic strategies. In terms of ovarian cell types, we found that oogonia, oocytes, granulosa cells, endothelial cells and immune cells identified in mammalian ovaries were also present in fish. The signature genes of these cell types were similar to those of other vertebrates (Fan et al., 2019). Additionally, we found that most of the DEGs of ovarian germ cells and granulosa cells identified in Chinese tongue sole are homologous to macaques, and these homologous genes are significantly enriched in biological processes or pathways related to their functions. These results suggest that although fish and mammals represent different reproduction models, the process of gamete production is conserved between these taxa.

TFs are crucial for the differentiation of oocytes. Differentially expressed TFs such as *arid3a*, *znf407*, *zeb1*, *pou2f2*, *ikzf2*, *tcf21*, *zhx1*, *gtf3ab,* and *znf706* were detected during early oogenesis, suggesting their possible roles in the development of germ cells. Two *gtf3a* gene paralogues (*gtf3aa* and *gtf3ab*) are present in teleost genomes due to the teleost-specific genome duplication event. *Gtf3ab* has been proved to be a marker of zebrafish oocyte differentiation, which facilitates massive oocyte stockpiling of 5S rRNAs. Notably, 5S rRNAs play essential roles in the developmental processes of newly formed embryos (Rojo-Bartolomé et al., 2020). Our data showed that *gtf3ab* was significantly upregulated from oogonia to oocytes, which suggests *gtf3ab* has the potential to serve as a broad marker of oocyte differentiation in diverse teleost species.

The granulosa cells of human follicles can be sorted into two functionally distinct subtypes: mural granulosa cells, which participate in cell differentiation and signal transduction; and cumulus cells, which participate in cell proliferation and metabolism (Harris et al., 2009; Roberts et al., 2002; Wigglesworth et al., 2015). In this study, we achieved the novel identification of five subtypes of follicle cells in Chinese tongue sole. Interestingly, we found that *foxl2* and *inhbb*, which have been proved to be expressed in human cumulus cells, were specifically expressed in GC1 (Pisarska et al., 2004; Fan et al., 2019; Sadat Tahajjodi et al., 2019). Therefore, we speculate that GC1 is a differentiated granulosa cell type in mature follicles and its function is similar to that of mammalian cumulus cells. Gene expression correlation analysis argued that GC2 is a proliferating granulosa cell subtype of GC1, as GC1 showed a strong correlation with GC2, and cell-cycle phase prediction analysis indicated that most of cells in GC2 were in G2/M phase (Supplementary Figures S2A, S2B). Interestingly, GC4 uniquely expressed *tnfaip6* whose orthologous gene in mammals is a signature gene in periovulatory cumulus cells playing a key role in the formation of the cumulus extracellular matrix (Fülöp et al., 2003). Notably, GC4 also specifically expressed *adamts1,* a gene required for the degradation of ECM in the cumulus oocyte complex to induce cumulus expansion and ovulation (Russell et al., 2003; Yang et al., 2021)*.* These argues that GC4 is a differentiated granulosa cell type in preovulatory follicles and plays a key role in ovulation. In contrast, *krt18*, a marker gene of pre-granulosa cells and mural granulosa cells in human ovary (Fan et al., 2019), was expressed in GC3. Additionally, GC3 showed specific high activity of TFs related to pre-granulosa cells, such as *lhx9*, *wt1,* and *irx3* (*Fu et al., 2020*; *Knarston et al., 2020*)*.* Accordingly, GC3 might be pre-granulosa cells surrounding early follicles. Nevertheless, we also observed the expression of ovulation associated genes *pgr* and *ptger4b* showed high specificity in GC3, and *pgr* showed high regulatory activity synchronously. *Pgr* and *ptger4b* have been proved to be highly expressed in the preovulatory follicles of medaka to regulate the ovulation process (Hagiwara et al., 2014). Therefore, we speculate that GC3 may be the source of other granulosa cells; however, it is not only a granulosa cell subtype in primordial follicles, but has a broader function at any stage of oocytes, which is inconsistent with that observed in mammals. One problem is whether these different states of follicle cells are assigned to different cell types or different developmental stages. Our results are more inclined to the former. First, the DEGs of these subtypes hardly overlap and showed differential functional enrichment. Second, we observed that the signature genes of mammalian cumulus cells and mural granulosa cells were differentially expressed in the subtypes. Third, ovulation associated genes are assigned to different subtypes, which indicates that ovulation requires the combined action of different granulosa cell subtypes. For theca cells, we only observed a single cell type, but distinct populations of theca cells exist in different follicles in humans (Fan et al., 2019); however, the function of theca cells (i.e., steroidogenesis) is conserved across vertebrates. From an evolutionary perspective, inconsistent follicular structures indicate the different investment strategies of parents in the reproductive process. These structures undergo diversification during evolution; however, the core function of the follicle cell population remains similar.

Niche–germline bidirectional communication coordinately regulates oogenesis, but the knowledge of the relevant signaling pathway interactions in fish remains limited. In this study, by conducting an unbiased ligand–receptor interaction analysis between the germ and somatic cell subclusters, we have identified many bidirectional communications between germ cells and granulosa cells, but less in theca cells, which may be ascribed to the spatial distribution of germ cells, granulosa cells and theca cells.

Additionally, we analysed the expression patterns of two common oogenesis-associated signaling pathways. We noted that, in Chinese tongue sole, *gdf9,* and *bmp15*, the ligand of the TGF-β signaling pathway involved in follicular development, were specifically expressed in germ cells. Moreover, *bmpr2*, the receptor of *bmp15*/*gdf9*, was expressed in germ cells and granulosa cells. These findings are consistent with observations in other vertebrates, i.e., human (Zhang et al., 2018), short finned eel and European sea bass (Halm et al., 2008; Falahati et al., 2021), suggesting that the function of *bmp15*/*gdf9-bmpr2* signaling in oogenesis is conserved across vertebrates. Moreover, our data revealed that the expression pattern of NOTCH signaling pathway components in fish was similar to that in human. This is consistent with the existing view that the NOTCH signaling pathway is highly conserved in vertebrates (Kopan and Ilagan, 2009).

Previous research demonstrated the ovulation mechanism of female mice, in which LH induces the expression of *PGR* and further stimulates granulosa cells to produce *adamts1*. Likewise, our study showed that *pgr* was specifically expressed in GC3, while *adamts1* was mainly expressed in GC4. Moreover, *ecm1*, encoding the target protein of *adamts1*, was expressed in all granulosa cell subtypes. Therefore, we speculate that the ovulation mechanism mediated by *adamts1* is conserved across vertebrates. Notably, *ptger4b*, a *pge2* receptor isoform of *ptger4*, has been proven to be crucial for ovulation in medaka (Fujimori et al., 2012; Hagiwara et al., 2014) and is expressed in pgr-dependent cells of zebrafish (Tang et al., 2016; Takahashi et al., 2018). Of particular interest was the finding that the expression patterns of *ptger4b* and *pgr* were consistent. The observations led to the proposal that *pge2*/*ptger4* signaling may have wide regulatory effects on ovulation across teleosts.

Based on the scRNA-seq method, this work provides several noteworthy contributions, as summarized below. We succeeded in identifying three germ cell types and five somatic cell types from fish ovary samples and revealed the gene expression signatures of each of these cell types. Especially, we identified four granulosa cell subtypes and one theca cell type in fish follicle, accompanied by different signature genes and transcriptional regulatory activities. Moreover, we found that the extensive niche–germline bidirectional communications regulate fish oogenesis, whereas ovulation in fish is accompanied by the coordination of simultaneous and tightly sequential processes across different granulosa cells. Altogether, these observations provide a global perspective on fish oogenesis, which may offer a valuable reference for future studies on fish resource propagation and contribute to the long-standing topics about the adaptability of reproductive strategies in lower vertebrates and common regularity of gametogenesis across vertebrates.

## Data Availability

According to national legislation/guidelines, specifically the Administrative Regulations of the People’s Republic of China on Human Genetic Resources (http://www.gov.cn/zhengce/content/2019-06/10/content_5398829.htm, http://english.www.gov.cn/policies/latest_releases/2019/06/10/content_281476708945462.htm), no additional raw data is available at this time. Data of this project can be accessed after an approval application to the China National Genebank (CNGB, https://db.cngb.org/cnsa/). Please refer to https://db.cngb.org/, or email: CNGBdb@cngb.org for detailed application guidance. The accession code CNP0002319 should be included in the application.
