# Supplementary figures and images for: Single-Cell Atlas of the Chinese Tongue Sole (Cynoglossus semilaevis) Ovary Reveals Transcriptional Programs of Oogenesis in Fish
Source: Front Cell Dev Biol. 2022 Mar 1;10:828124. doi: 10.3389/fcell.2022.828124 (PMC8921555; doi:10.3389/fcell.2022.828124)

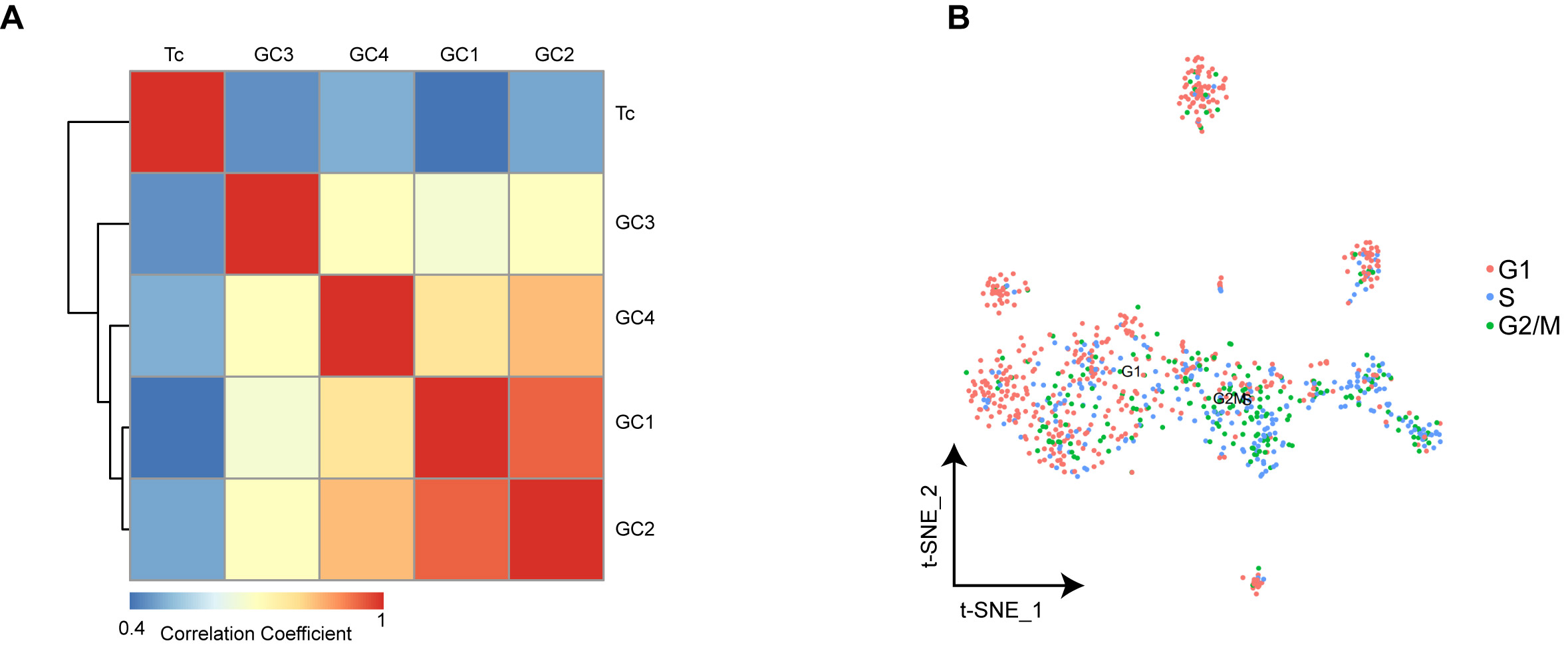

Supplement: Supplementary file 3 [file Image2.JPEG]

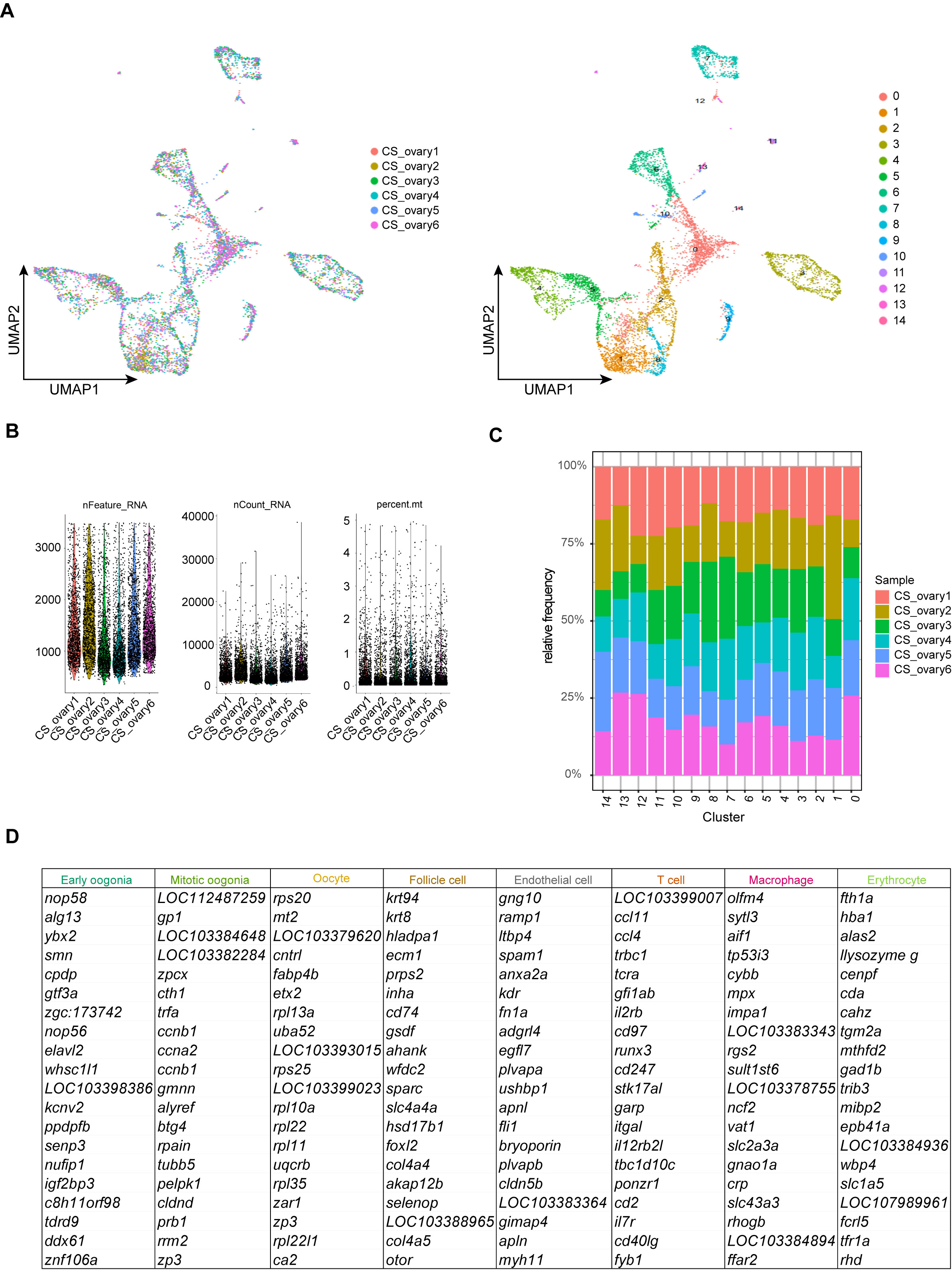

Supplement: Supplementary file 6 [file Image1.jpg]
